# Supplementary material for: Association between oral microbiome and seven types of cancers in East Asian population: a two-sample Mendelian randomization analysis
Source: Front Mol Biosci. 2023 Nov 21;10:1327893. doi: 10.3389/fmolb.2023.1327893 (PMC10702768; doi:10.3389/fmolb.2023.1327893)
Supplement: Supplementary file 1 [file DataSheet1.ZIP › Supplementary Materials/MR plots of saliva/saliva═╝/Lung Cancer/pheno.388_to_lung cancer_forest.pdf]

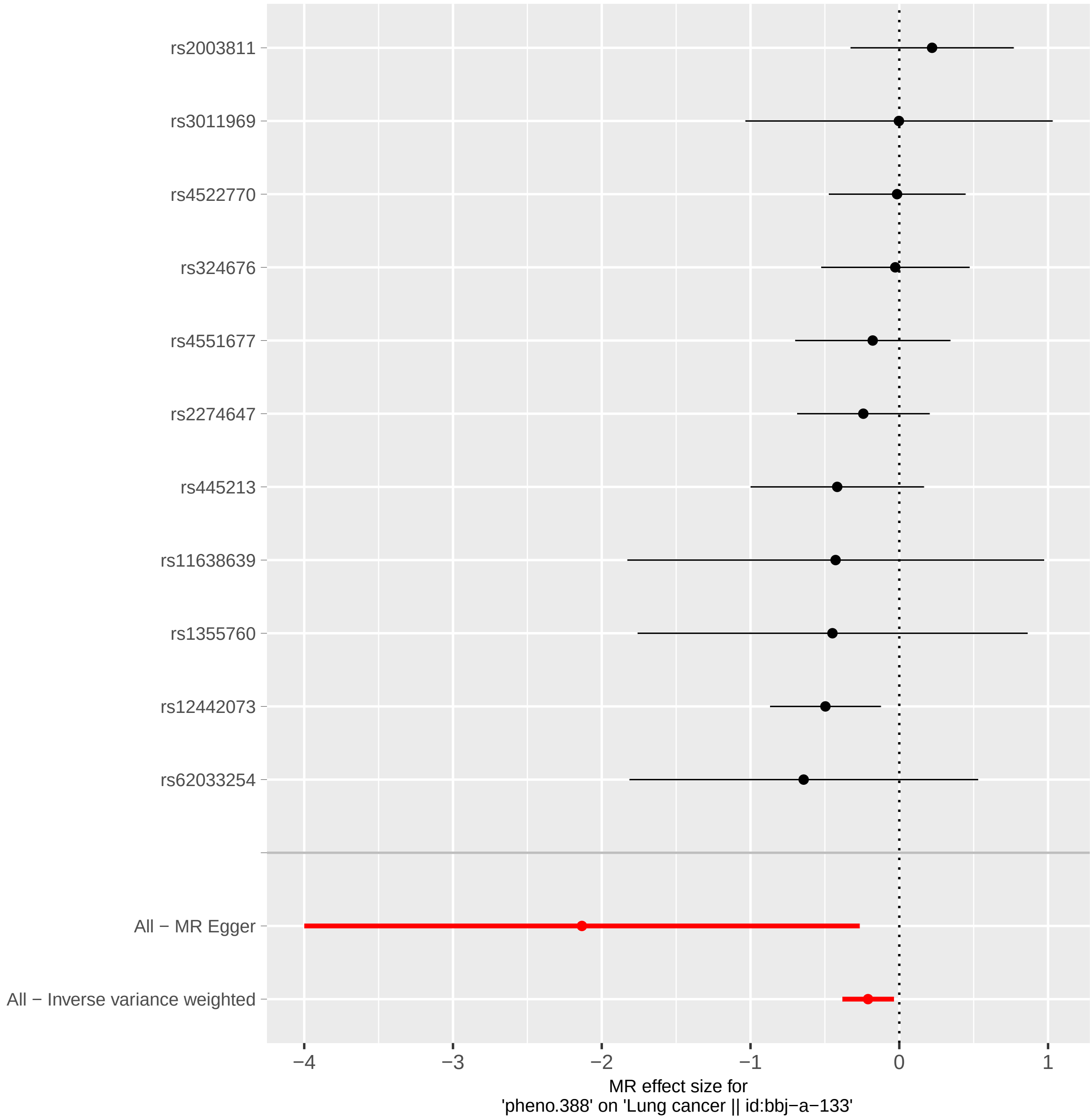

rs2003811
rs3011969
rs4522770
rs324676
rs4551677
rs2274647
rs445213
rs11638639
rs1355760
rs12442073
rs62033254
All − MR Egger
All − Inverse variance weighted
−4
−3
−2
−1
0
1
MR effect size for
'pheno.388' on 'Lung cancer || id:bbj−a−133'
